# Commentary on: Immersive robotic colorectal training in the United Kingdom is safe and efficient

**DOI:** 10.1308/rcsann.2026.0009

**Published:** 2026-03-18

**Authors:** S Vijayaraghavalu

**Affiliations:** George Eliot Hospital NHS Trust, UK

I read with great interest the article by Franklyn *et al* evaluating the outcomes of an immersive robotic colorectal fellowship in the United Kingdom (UK).^1^ As robotic platforms become increasingly integrated into colorectal practice, structured and efficient training models are urgently needed. The authors should be commended for presenting evidence that high-intensity, ring-fenced fellowships can deliver safe, oncologically sound outcomes without compromising operative efficiency.

Their finding that fellows achieved comparable postoperative and oncological results to consultant surgeons is particularly significant. The comparable lymph node yield, margin positivity and postoperative morbidity mirror observations from other robotic training programmes, suggesting that structured modular training and supervised console time enable rapid progression to competence.^2,3^

The shorter operative times observed in the fellow cohort further challenge the common concern that robotic training slows theatre productivity. This aligns with existing data showing that concentrated exposure and consistent case volume are associated with faster skill acquisition than protracted, sporadic training models.^4^

Importantly, the study highlights the ongoing training imbalance created by ring-fenced robotic lists, an issue repeatedly raised by UK surgical trainees. As robotics expands, the development of nationally standardised pathways – integrated within the surgical curriculum – will be essential to ensure equitable exposure for all trainees and to prevent marginalisation of registrars during high-intensity fellowships.^5^

Overall, this work provides valuable evidence that immersive robotic colorectal training can safely and efficiently prepare post-Certificate of Completion of Training trainees for independent practice. The model described has clear implications for shaping future UK-wide robotic training structures.

## Competing interests

The author declare no competing interests.

## Funding

The author received no financial support for the research, authorship, and/or publication of this article.

## Author contributions

**S Vijayaraghavalu**: Conceptualisation, Writing – original draft, Writing – review & editing.

## Artificial Intelligence

The author/s declare that no AI was used to conduct the study or prepare the manuscript.
